# Association of low birth weight with undernutrition in preschool-aged children in Malawi

**DOI:** 10.1186/s12937-019-0477-8

**Published:** 2019-09-02

**Authors:** Peter Austin Morton Ntenda

**Affiliations:** School of Public Health and Family Medicine, Department of Public Health, College of Medicine, University of Malawi, Private Bag 360, Chichiri, Blantyre, 3 Malawi

**Keywords:** Birthweight, Undernutrition, Low birth weight, Child health, Malawi

## Abstract

**Background:**

Malnutrition refers to deficiencies, excesses, or imbalances in a person’s intake of energy and/or nutrients. The term malnutrition is a broad term encompassing the three conditions namely undernutrition (micronutrient-related malnutrition), over-nutrition (overweight and obesity), and diet-related non-communicable diseases. Undernutrition is defined as the outcome of insufficient food intake and repeated infectious diseases. Low birth weight (LBW) is cited as a risk factor for mortality and morbidity in young children. However, its association with undernutrition has received little attention. Thus, the current study aimed to examine the relationship between LBW and undernutrition in Malawi.

**Methods:**

A cross-sectional study was conducted using data from the Malawi Demographic and Health Survey (MDHS) 2015–16. Children whose Z-scores for each of the following indices height-for-age, weight-for-height, and weight-for-age were below the minus two standard deviations (−2SD) from the median of the World Health Organization’s (WHO) reference population were considered to be stunted, wasted and underweight, respectively. LBW was defined as babies whose birth weight was less than 2500 g. The multivariate logistic regression models were performed using surveylogistic while controlling various confounding factors in the six different models.

**Results:**

The prevalence of stunted, underweight, wasted, and LBW were reported as follows, 39%. 11, 2, and 10% respectively. Compared to children with normal/average birth weight, those with LBW had significantly higher odds of being stunted [adjusted odds ratio (aOR): 1.72; 95% confidence interval (CI): 1.35–2.20), underweight (aOR: 2.30; 95% CI: 1.68–3.14) and wasted (aOR: 1.47; 95% CI: 1.38–4.25) respectively.

**Conclusions:**

LBW was a strong predictor of all the three indices of undernutrition. Interventions that aim at improving the growth and development of children during the early years should consider addressing factors that trigger LBW.

**Electronic supplementary material:**

The online version of this article (10.1186/s12937-019-0477-8) contains supplementary material, which is available to authorized users.

## Background

Globally, malnutrition is one of the leading causes of morbidity and mortality in preschool-aged children [1–3]. Childhood malnutrition is known to have both acute and chronic consequences that are harmful to the growth and development of pre-school children [2, 3]. Malnutrition refers to deficiencies, excesses, or imbalances in a person’s intake of energy and/or nutrients. Malnutrition is a broad term that describes the three conditions, namely undernutrition including micronutrient-related malnutrition, over-nutrition (overweight and obesity), and diet-related non-communicable diseases [4]. By the end of 2017, the World Health Organization (WHO) estimated that 462 million children below 5 years of age had low weight for their age, whilst 155 million had low height for their age, and 52 million had low weight for their heights [4]. It is known that about half of all mortality in preschool-aged children are due to poor nutrition and the burden is prominent in South Asia and sub-Saharan Africa [2–5]. The causes of undernutrition are complex, multifactorial and poorly understood, however, the United Nations Children’s Fund (UNICEF) stated that the immediate causes of malnutrition are the inadequate dietary intakes and frequent illnesses [6, 7].

Nutrition constraints remain throughout the life cycle of the human being [8, 9]. Specifically, the lack of good and balanced nutrition time and again begins in utero among women with poor nutritional status before and in the course of pregnancy and extends throughout the life course. Predominantly, the burden of malnutrition is more pervasive in girls and women through adolescent and adult life [10]. Unfortunately, poor nutrition that occurs in the course of childhood, adolescence, and pregnancy has a negative influence on the birth outcomes, including low birth weight (LBW) [11]. As such, babies who had experienced intrauterine growth retardation (IUGR), have a high risk of being undernourished at birth as well as have a higher likelihood of dying in the neonatal period or later in infancy [11]. Conversely, if those infants progress in life, they do not only have problems in catching up on the lost growth, but are also more likely to experience a variety of developmental problems. Thus, LBW infants have a higher risk of becoming underweight or stunted in early life [11]. Thus, good nutrition is critical for infants’ optimal growth and development and has an essential role in shaping their health later in life. Globally, in 2013, the UNICEF reported that approximately 16% of babies had LBW [12]. Corresponding figures from the WHO revealed that about 14% of LBW infants resided in Africa [13]. Whereas in Malawi, the prevalence of LBW was estimated at 12%, which is close to those figures estimated at global level as well as in Africa [14]. The goal of the WHO is to achieve a 30% decrease in the number of newborn with a weight lower than 2500 g by the year 2025 [15, 16].

Despite the long-standing efforts made by the Malawi government [17] to combat poor nutrition in children under 5 years of age, the rates of malnutrition remain unacceptably high [14, 18, 19]. According to the 2016 national estimates on childhood undernutrition in Malawi, 37% of young children were stunted, 3% were wasted, and 12% were underweight [14]. In many settings, LBW has been reported as a risk factor for mortality and morbidity in young children [20–22]. Even though a number of studies classified LBW as a potential risk factor for malnutrition [23–25], most of these studies did not consider LBW as the main risk factor of importance. Therefore, it remains unknown how and to what extent LBW is related to childhood malnutrition during the early years of life. Given that a certain proportion of children born with LBW (12.3%) in Malawi [14], the current study was conducted to establish an association between LBW and childhood undernutrition in Malawi.

## Methods

### Data source

The current study utilized a population-based cross-sectional data taken from the Malawi Demographic and Health Survey (MDHS) 2015–16 which was conducted between October 2015 and February 2016. The procedures used in this study can be found in details elsewhere [14]. In brief, the MDHS was designed to yield a nationally representative sample at the national level, residence level (urban-rural) and the regional level). The survey employed a stratified two-stage cluster design. In the first stage, the country was stratified depending upon the place of residence as well as subnational administrative regions to construct stratum. Using the probability proportional to size technique, a certain number of enumeration areas (EAs) from each stratum are drawn. The population size was drawn from Census files. In the second stage, a fixed number of households were selected from EAs using the systematic random sampling technique. In the 2015–16 MDHS, a total of 27,516 households were selected for the sample, of which 26,564 were occupied. Of the occupied households, 26,361 were successfully interviewed, for a response rate of 99%. Secondly, in the interviewed households, 25,146 eligible women were identified for individual interviews. However, interviews were completed with 24,562 women, for a response rate of 98%.

### Data collection

The survey collected weight and height of preschool-aged children in a sub-sample of one-third of the households, regardless of whether their mothers got interviewed or not. Thus, a total of 6033 children under age 5 were eligible for height and weight measurements. The analysis of height-for-age indices include 94% of eligible children with complete and valid height measurement and age data. Analysis of weight-for-height indices includes 95% of eligible children with complete and valid height and weight measurements. Analysis of weight-for-age indices includes 96% of eligible children with complete and valid weight measurement and age data. Thus, the final sample analyzed by the MDHS was 5786. An electronic SECA 878 flat scale was used to collect the weight measurements. Whilst a Shorr Board® measuring board was used to estimate the height measurements [14]. Recumbent lengths were measured for children less than 2 years old while standing heights were measured for children aged 24 months or older [14]. The data on height and weight were used to calculate several measures of childhood nutritional status, namely; height-for-age, weight-for-height, and weight-for-age. The socio-demographic information was collected from women of reproductive age (15–49 years) who had children below 5 years prior to the survey by means of personal interviews.

### Inclusion and exclusion

The current study selected children of age group 6–59 months. Children who were missing measurements on birth weight, weight and height were excluded from the analysis. Children whose households were not sampled for height and weight measurements as well as who had missing data on other covariates were also excluded. In addition, this study only included children whose caregivers had been interviewed. After applying the inclusion and exclusion criteria, which included age restriction, 4047 children under 5 years old were analyzed in this study.

## Study variables

### Dependent variable

Childhood undernutrition was the dependent variable of the present study, and the variable was further disintegrated into three indices, namely stunting (low-height-for-age), underweight (low-weight-for-age) and, wasting (low-weight-for-height) [14, 19]. Each of these indices is expressed as Z-score from the median of the WHO Child Growth Standards reference population [26]. Particularly, children whose Z-scores for any of these three indices were below minus two standard deviations (−2SD) from the median of the WHO’s reference population were considered as stunted, wasted or underweight, respectively [26]. Stunting reflects the linear growth achieved during the pre- and post-natal periods with its deficits indicating long-term, cumulative effects of inadequate nutrition and/or health [27]. Wasting indicates acute or current malnutrition that is the consequence of the poor dietary intake or frequent occurrence of infectious disease [28]. Underweight is influenced by the height and weight of the child and thus it’s a combination of stunting and wasting [28].

### Main independent variables

The main dependent variable of the current study was birth weight – expressed in grams (g). According to the WHO and UNICEF, birth weight is defined as the first weight of the live newborn or fetus collected soon after birth [29, 30]. The MDHS 2015–16 reported that birth weight was recorded from either a written record (child’s health cards) or mother’s recall. Thus, to limit recall bias, the present study included only children who had valid birth weight recorded in their health cards. All children who did not have valid birth weight recorded in their health cards, children who were not weighed, whose delivery occur in homes or other places were excluded. Using the guidelines set by WHO and UNICEF, the birth weight was categorized as LBW or normal birth weight. LBW was defined as birth weight below 2500 g i.e. up to and including 2499 g. On the other hand, greater than or equal 2500 g was classified as normal birth weight [30].

### Covariates

It is known that child undernutrition is the result of multiple and interrelated factors. Therefore, apart from the LBW various control variables were included in the present study following careful review of the relevant literature [3, 31], and were grouped as child biological factors, child health factors, maternal/household factors, household environmental factors, health service and seeking behavior related factors, and community level factors (Table 1). Child biological factors include child’s sex (male/female) child’s age (< 12 months /12–23 months/24–35 months /35–47 months/48–59), type of birth (single births/multiple births), preceding birth interval (< 24 months/12–47 months/48+ months). Child health factors recent diarrhea (no/yes) and recent fever (no/yes). Maternal factors include, women’s age (15–19 years/20–24 years/25–29 years/ 30–34 years/35–49 years), women’s education (none/primary/secondary education and higher), body mass index (underweight/ optimal weight/ overweight/or obesity), wealth index (poorest/ poorer/ middle/richer/ richest), number of under-5-year children in the household (≤1/2–3/4+), and amount of media exposure (0/1/2/3). Household environmental factors include the source of drinking water (unimproved/ improved) and sanitation facility (unimproved/improved). Health Service utilization factors include the place of delivery (home and other/hospital/institution) and perceived distance to the health facility (not perceived to be a problem or perceived problem). Community characteristics include place of residence (rural/urban) and region (northern/central/ southern). The household wealth index was generated using a statistical method well-known as principal components analysis (PCA) by the Monitoring and Evaluation to Assess and Use Results Demographic and Health Surveys (MEASURE DHS) [32]. Amount of media exposure was derived from three items namely frequency of reading a newspaper, watching television, and or listening to radio at once a week. The MDHS asked respondents the following questions; (1) Do you read a newspaper or magazine at least once a week, less than once a week or not at all? (2) Do you listen to the radio at least once a week, less than once a week or not at all? And (3) Do you watch television at least once a week, less than once a week or not at all? [14]. Then, the amount of media exposure was calculated by summing up the reported frequency of each media if an activity was performed at least once a week. Furthermore, diarrhea was defined as the passage of three or more loose or liquid stools during a 24-h period while fever was determined by self-reports made by caregivers about the symptoms that had occurred within 2 weeks prior to the survey [14]. Specifically, caregivers were asked; has (NAME) been ill with a fever at any time in the last 2 weeks? And has (NAME) had diarrhea in the last 2 weeks?

**Table 1:** Baseline characteristics of the study participants MDHS, 2015–16 (*n* = 4047)

Characteristic	Overall sample n^k^ (%)^l^	Stunted %	Underweight %	Wasted %
Outcome variables^a^				
Stunted				
No	2521 (61.03)	–	–	–
Yes	1526 (38.97)	–	–	–
Underweight				
No	3551 (88.69)	–	–	–
yes	496 (11.31)	–	–	–
Wasted				
No	3933 (97.70)	–	–	–
Yes	114 (2.30)	–	–	–
Main independent variable				
Birth weight				
Low birthweight^b^	399 (10.11)	50.63*	23.81*	5.26*
Normal and above	3648 (89.89)	36.29	10.99	2.55
Child characteristics				
Sex of the child				
Male	2005 (48.78)	39.30*	12.87	3.09
Female	2042 (51.22)	36.14	11.66	2.55
Age				
6–11	459 (11.70)	22.88*	9.59*	4.58*
12–23	926 (21.98)	38.88	11.45	3.46
24–35	921 (22.61)	41.91	12.49	2.71
36–47	905 (22.98)	43.54	14.92	2.10
48–59	836 (20.73)	33.61	11.48	2.03
Multiple births				
Single births	3927 (97.09)	37.08*	12.12	2.85
Twins	120 (2.91)	58.33	16.67	1.67
Preceding birth interval (months)				
< 24	1352 (32.50)	40.24*	13.83	3.55
24–47	1624 (41.30)	37.93	11.88	2.46
48+	1071 (26.20)	34.17	10.83	2.43
Child health related factors				
Recent diarrhea^c^				
No	3178 (78.09)	37.44	11.80	2.39*
Yes	869 (21.91)	38.67	13.92	4.37
Recent fever^d^				
No	2783 (68.01)	37.33	11.93	2.34*
Yes	1264 (31.99)	38.53	12.97	3.88
Maternal/household Characteristics				
Maternal age (years)				
15–19	239 (6.13)	37.24*	10.88	4.60
20–24	1225 (29.36)	41.80	14.37	3.27
25–29	1008 (24.62)	35.12	10.42	2.78
30–34	792 (20.27)	34.22	11.74	2.15
35–49	783 (19.63)	38.31	12.26	2.30
Maternal education levels				
No education	542 (14.16)	46.13*	14.21*	2.03
Primary	2685 (67.47)	38.40	12.63	2.83
Secondary and above	820 (18.37)	29.88	9.76	3.29
Maternal body mass index^e^				
Underweight	229 (5.62)	43.67*	20.09*	5.68*
Optimal weight	3014 (75.00)	39.38	12.67	2.92
Overweight/obesity	804 (19.32)	29.73	8.46	1.62
Wealth index				
Poorest	955 (26.09)	44.61*	15.39*	3.35
Poorer	958 (24.69)	41.96	13.78	2.51
Middle	800 (19.34)	38.50	11.50	2.63
Richer	729 (17.55)	31.96	10.01	2.47
Richest	605 (12.34)	25.95	8.60	3.14
No of under-5-year children				
≤ 1	1938 (47.51)	35.04*	11.56*	2.53*
2–3	1776 (44.29)	39.36	11.88	2.87
≥ 4	333 (8.20)	44.44	18.32	4.20
Amount of media exposure^f^				
0	2621 (64.76)	40.02*	13.47*	3.13
1	1112 (27.48)	35.25	10.97	1.98
2	272 (6.72)	27.57	6.62	2.94
3	42 (1.04)	23.81	7.14	4.76
Household environmental factors				
Source of drinking water				
Unimproved	670 (15.88)	41.34*	14.18	3.13
Improved^g^	3377 (84.12)	36.99	11.87	2.75
Sanitation facility				
Unimproved	806 (20.80)	39.70	11.91	2.48
Improved^h^	3241 (79.20)	37.21	12.34	2.90
Health Service utilization				
Place of delivery				
Home and other	335 (8.79)	40.30	17.01*	3.28
Hospital/institution^i^	3712 (91.21)	37.47	11.83	2.77
Perceived distance to health facility				
Perceived problem	1773 (40.28)	37.79	12.35	2.88
Not perceived problem	2274 (59.72)	37.64	12.18	2.77
Community characteristics				
Place of residence				
Urban	481 (7.91)	27.86*	10.40	4.37*
Rural	3566 (92.09)	39.04	12.51	2.61
Geographical region				
Northern	710 (10.81)	31.69*	10.00	2.11
Central	1458 (43.18)	40.26	11.93	2.40
Southern	1879 (46.01)	38.00	13.36	3.41

^a^Each index is expressed in SD from the median of the WHO Child Growth Standards; *SD* standard deviation, *WHO* World Health Organization; ^b^Proportion of births with a reported birth weight < 2500 g regardless of gestational age; ^c^passage of three or more loose or liquid stools during a 24-h period; ^d^self-reports by mothers about symptoms that had occurred within 2 weeks prior to the survey; ^e^weight in kilograms divided by the square of his height in meters (kg/m^2^); ^f^frequency of reading newspaper or magazine, frequency of listening to radio, frequency of watching television.; ^g^improved drinking water (piped water into dwelling, piped water to yard/plot, public tap or standpipe, tubewell or borehole, protected dug well, protected spring and rainwater); ^h^improved sanitation (flush toilet, piped sewer system, septic tank, flush/pour flush to pit latrine, ventilated improved pit latrine, pit latrine with slab, and composting toilet); ^i^government hospital, government health center, government health post/outreach, other public sector, private hospital/clinic, CHAM/MISSION hospital, CHAM/MISSION health Center, BLM, other private medical sector, * < 0.05; ^**k**^Unweighted frequency; ^**l**^Weighted percentage

## Data analysis

All data analyses were performed using the statistical analysis software (SAS) for Windows, version 9.4 (SAS Institute, Cary, NC, USA). The survey-specific SAS procedures PROC SURVEYFREQ and PROC SURVEYLOGISTIC for weighting, clustering, and stratification were applied taking into account the hierarchal nature of the survey design [33]. Baseline characteristics were summarized as frequency, weighted percentage, and standard errors. The multivariate analyses were conducted by means of the logistic regression to estimate the effects of the birth weight on the childhood undernutrition while adjusting for six models, namely child’s biological, child’s health, maternal/households, household environmental, healthcare utilization and community characteristics). Adjusted odds ratios (aORs) and 95% confidence intervals (CIs) with their *P*-values were calculated. *P*-values of less than 0.05 were considered as statistical significance. The multicollinearity was tested by the use of variance inflation factor and tolerance. The results of collinearity shown no multicollinearity existed in the models (Additional file 1: Table S1). Additional file 2: Figure S1 shows the fit diagnostics for childhood undernutrition, which showed that a lot of variations in the models (stunting, underweight, and wasting) were unexplained (results from the proportion less).

## Ethical statement

The procedures and questionnaires for MDHS 2015–16 were reviewed and approved by the Malawi National Health Sciences Research Committee (NHSRC) and the Institutional Review Board (IRB) of ICF Macro. ICF IRB ensured that the survey complied with the U.S. Department of Health and Human Services regulations for the protection of human subjects (45 CFR 46) [34], while the NHSRC ensured that the survey was conducted in line with Malawian laws and norms [14]. At the beginning of each interview, participants were asked to give verbal and written consent (informed consent). The informed consent statement emphasizes that participation was voluntary and that the respondent may opt to refuse to answer any question. Furthermore, the authors sought permission from the DHS program for the use of the data beyond the primary purpose it was collected.

## Results

### Baseline characteristics of the study population

A total of 4047 children under 5 years were sampled and analyzed in this study. Baseline characteristics of the study population are presented in Table 1. Overall, approximately 39% of children were stunted, while 12 and 2% were underweight and wasted respectively. In terms of birth weight, a majority of children (about 90%) were born with normal and above weight. With regards to child biological and health-related factors, more than half (51%) of the children were female and a majority of them (97%) were the products of single births while 41% of children born between 24 and 47 month intervals. Furthermore, about 22 and 32% of children had diarrhea and fever episodes in the last 2 weeks respectively. In terms of maternal and household characteristics, approximately 29% of respondents were distributed in the age group 20–24 years, two-thirds had primary school education and a majority (75%), had a normal body mass index. Additionally, only 1% of the participants were exposed to all forms of media (newspaper, radio, and Television). With regards to household environmental and health service utilization factors, a majority of participants 84%, had access to an improved water source while nearly 80% had improved sanitation facilities. Similarly, a majority of participants (> 90%) had institutional delivery, whilst 59% had perceived the distance to the nearest health facility to a big problem. In terms of community characteristics, 46% of respondents were southern region dwellers and 92% were rural dwellers.

### Prevalence of undernutrition according to sociodemographic factors

Table 1, also presents the prevalence of stunting, underweight, and wasting by sociodemographic characteristics. The prevalence of stunting was observed to be significantly high in children with a low birth weight, male children, in children aged 36–47 months, in children who were products of multiple births, in children whose proceeding birth intervals were less than 24 months, inn children whose mothers were 35–49 years, in children whose mothers had no formal education, in children whose mothers had BMI less than 18.5 kg/m^2^, in children who were residing in the poorest households, in children whose households had ≥4 under-five children, in children whose households were not exposed to mass media, form in children whose households had no access to improved water sources, in children from rural areas, and in children from central region.

With respect to underweight, the prevalence was observed to be significant high in children with a low birthweight, in children aged 36–47 months, in children whose mothers had no formal education, in children whose mothers had BMI less than 18.5 kg/m2, in children who were residing in the poorest households, in children whose households had ≥4 under-five children, in children whose households were not exposed to mass media, and in children who delivered occurred in homes and other places.

In terms of wasting, the prevalence was observed to be significant high in children with a low birthweight, in children aged 6–11 months, in children with an episode of fever in the last 2 weeks, in children with an episode of diarrhea in the last 2 weeks, in children whose mothers had BMI less than 18.5 kg/m2, in children whose households had ≥4 under-five children, and in children from rural areas.

### Low birthweight and the risk of childhood undernutrition

Tables 2, 3 and 4 present the association of the LBW with childhood undernutrition. The unadjusted models (Model 1) showed that compared to children who had normal birth weight those with LBW had an increased odds of being stunted (Crude odds ratio [CrOR]:1.72; 95% confidence interval [CI]: 1.35–2.20), of being underweight (CrOR: 2.30; 95% CI: 1.68–3.14), and, of being wasted (CrOR: 2.42; 95% CI: 1.38–4.25). The associations remain unchanged even after adjustments for the child’s biological factors (Model 2), child’s health factors (Model 3), maternal or household factors (Model 4), household environmental factors (Model 5), and health service utilization (Model 6) though with varying strength of odd ratios. The full models (Model 7) showed that the odds increased by 55% for being stunted (adjusted odds ratio [aOR]: 1.55; 95% CI: 1.91–2.01), 16% for being underweight (aOR: 2.16; 95% CI: 1.56–2.99), and 51% for being wasted (aOR: 2.53; 95% CI: 1.40–4.57) in children who had LBW compared to those children who were born with normal weight.

**Table 2:** Multivariate logistic analysis of the association of low birthweight with childhood stunting while controlling for several covariates

Variables	Model I	Model II	Model III	Model IV	Model V	Model VI	Model VII
	COR 95% (CI)	aOR 95% (CI)	aOR 95% (CI)	aOR 95% (CI)	aOR 95% (CI)	aOR 95% (CI)	aOR 95% (CI)
Birth weight							
Low birthweight^a^	1.72 (1.35–2.20)*	1.63 (1.26–2.10)*	1.63 (1.26–2.11)*	1.55 (1.19–2.01)*	1.55 (1.19–2.01)*	1.54 (1.18–2.00)*	1.55 (1.19–2.01)*
Normal and above	1.00	1.00	1.00	1.00	1.00	1.00	1.00
Sex of the child							
Male		1.21 (1.04–1.41)*	1.21 (1.04–1.41)*	1.22 (1.05–1.43)*	1.22 (1.05–1.43)*	1.23 (1.05–1.43)*	1.23 (1.05–1.44)*
Female		1.00	1.00	1.00	1.00	1.00	1.00
Age							
6–11		0.56 (0.41–0.76)*	0.54 (0.39–0.74)*	0.50 (0.36–0.69)*	0.49 (0.36–0.69)*	0.49 (0.35–0.69)*	0.49 (0.36–0.69)*
12–23		1.19 (0.93–1.51)	1.16 (0.90–1.48)	1.08 (0.84–1.39)	1.08 (0.84–1.39)	1.08 (0.84–1.39)	1.08 (0.84–1.39)
24–35		1.46 (1.14–1.86)*	1.45 (1.13–1.86)*	1.39 (1.08–1.78)*	1.39 (1.08–1.78)*	1.38 (1.08–1.77)*	1.39 (1.09–1.78)*
36–47		1.54 (1.21–1.95)*	1.53 (1.21–1.95)*	1.52 (1.20–1.93)*	1.52 (1.20–1.93)*	1.52 (1.20–1.93)*	1.53 (1.20–1.94)*
48–59		1.00	1.00	1.00	1.00	1.00	1.00
Multiple births							
Single births		0.49 (0.28–0.88)*	0.50 (0.28–0.89)*	0.47 (0.27–0.81)*	0.47 (0.28–0.82)*	0.48 (0.28–0.83)*	0.47 (0.28–0.81)*
Twins		1.00	1.00	1.00	1.00	1.00	1.00
Preceding birth interval							
< 24		1.23 (0.99–1.53)	1.23 (0.99–1.53)	1.05 (0.80–1.39)	1.05 (0.80–1.39)	1.05 (0.80–1.39)	1.04 (0.79–1.37)
24–47		1.14 (0.91–1.42)	1.14 (0.91–1.42)	0.95 (0.74–1.20)	0.94 (0.74–1.20)	0.95 (0.74–1.20)	0.93 (0.73–1.18)
48+		1.00	1.00	1.00	1.00	1.00	1.00
Recent diarrhea^b^							
No			0.90 (0.73–1.11)	0.87 (0.70–1.08)	0.87 (0.70–1.07)	0.87 (0.71–1.08)	0.87 (0.70–1.08)
Yes			1.00	1.00	1.00	1.00	1.00
Recent fever^c^							
No			1.05 (0.89–1.26)	1.08 (0.90–1.29)	1.08 (0.90–1.29)	1.07 (0.90–1.28)	1.07 (0.90–1.28)
Yes			1.00	1.00	1.00	1.00	1.00
Maternal age							
15–19				0.86 (0.56–1.31)	0.86 (0.56–1.31)	0.84 (0.55–1.29)	0.87 (0.57–1.33)
20–24				1.15 (0.86–1.54)	1.15 (0.86–1.53)	1.14 (0.85–1.52)	1.16 (0.87–1.55)
25–29				0.91 (0.69–1.21)	0.91 (0.69–1.21)	0.90 (0.68–1.20)	0.91 (0.69–1.20)
30–34				0.85 (0.63–1.13)	0.85 (0.63–1.13)	0.84 (0.62–1.12)	0.85 (0.63–1.13)
35–49				1.00	1.00	1.00	1.00
Maternal education levels							
No education				1.56 (1.13–2.18)*	1.57 (1.13–2.17)*	1.58 (1.14–2.20)*	1.59 (1.15–2.21)*
Primary				1.17 (0.93–1.46)	1.17 (0.93–1.46)	1.17 (0.93–1.46)	1.15 (0.92–1.44)
Secondary and above				1.00	1.00	1.00	1.00
Maternal body mass index^d^							
Underweight				2.02 (1.34–3.04)*	2.03 (1.34–3.06)*	2.05 (1.36–3.09)*	2.06 (1.36–3.10)*
Optimal weight				1.46 (1.16–1.84)*	1.46 (1.16–1.84)*	1.48 (1.17–1.86)*	1.47 (1.16–1.85)*
Overweight/obesity				1.00	1.00	1.00	1.00
Wealth index							
Poorest				2.28 (1.64–3.19)*	2.30 (1.64–3.24)*	2.36 (1.67–3.33)*	2.04 (1.40–2.98)*
Poorer				2.07 (1.48–2.90)*	2.08 (1.48–2.92)*	2.13 (1.50–3.01)*	1.84 (1.25–2.69)*
Middle				1.73 (1.25–2.40)*	1.73 (1.24–2.41)*	1.76 (1.26–2.46)*	1.55 (1.08–2.24)*
Richer				1.29 (0.91–1.82)	1.29 (0.91–1.83)	1.32 (0.93–1.87)	1.18 (0.81–1.72)
Richest				1.00	1.00	1.00	1.00
No of under-5-year children							
≤ 1				0.71 (0.53–0.96)*	0.71 (0.53–0.96)*	0.70 (0.52–0.95)*	0.70 (0.52–0.95)*
2–3				0.78 (0.59–1.05)	0.78 (0.59–1.05)	0.78 (0.58–1.05)	0.79 (0.59–1.05)
≥ 4				1.00	1.00	1.00	1.00
Amount of media exposure^e^							
0				0.64 (0.20–2.03)	0.64 (0.20–2.04)	0.66 (0.20–2.12)	0.64 (0.21–1.99)
1				0.66 (0.21–2.11)	0.67 (0.21–2.12)	0.69 (0.21–2.20)	0.65 (0.21–2.02)
2				0.53 (0.17–1.71)	0.54 (0.17–1.72)	0.54 (0.17–1.74)	0.53 (0.17–1.67)
3				1.00	1.00	1.00	1.00
Source of drinking water							
Unimproved					1.02 (0.82–1.28)	1.04 (0.83–1.30)	1.03 (0.82–1.29)
Improved^f^					1.00	1.00	1.00
Sanitation facility							
Unimproved					0.95 (0.78–1.15)	0.95 (0.78–1.16)	0.95 (0.78–1.16)
Improved^g^					1.00	1.00	1.00
Place of delivery							
Home and other						0.90 (0.68–1.20)	0.90 (0.68–1.20)
Hospital/institution^h^						1.00	1.00
Perceived Distance to health facility							
Perceived problem						1.13 (0.96–1.34)	1.15 (0.97–1.36)
Not perceived problem						1.00	1.00
Place of residence							
Urban							0.70 (0.50–0.99)*
Rural							1.00
Geographical region							
Northern							1.07 (0.85–1.36)
Central							1.18 (0.98–1.42)
Southern							1.00

*P < 0.05; ^a^Proportion of births with a reported birth weight < 2500 g regardless of gestational age; ^b^passage of three or more loose or liquid stools during a 24-h period; ^c^self-reports by mothers about symptoms that had occurred within 2 weeks prior to the survey; ^d^weight in kilograms divided by the square of his height in meters (kg/m^2^); ^e^frequency of reading newspaper or magazine, frequency of listening to radio, frequency of watching television.; ^f^improved drinking water (piped water into dwelling, piped water to yard/plot, public tap or standpipe, tubewell or borehole, protected dug well, protected spring and rainwater); ^g^improved sanitation (flush toilet, piped sewer system, septic tank, flush/pour flush to pit latrine, ventilated improved pit latrine, pit latrine with slab, and composting toilet); ^h^government hospital, government health center, government health post/outreach, other public sector, private hospital/clinic, CHAM/MISSION hospital, CHAM/MISSION health Center, BLM, other private medical sector

**Table 3:** Multivariate logistic analysis of the association of low birthweight with childhood underweight while controlling for several covariates

Variables	Model I	Model II	Model III	Model IV	Model V	Model VI	Model VII
	COR 95% (CI)	aOR 95% (CI)	aOR 95% (CI)	aOR 95% (CI)	aOR 95% (CI)	aOR 95% (CI)	aOR 95% (CI)
Birth weight							
Low birthweight^a^	2.30 (1.68–3.14)*	2.28 (1.65–3.14)*	2.29 (1.65–3.16)*	2.15 (1.56–2.99)*	2.15 (1.55–2.98)*	2.15 (1.56–2.99)*	2.16 (1.56–2.99)*
Normal and above	1.00	1.00	1.00	1.00	1.00	1.00	1.00
Sex of the child							
Male		1.23 (0.99–1.52)	1.22 (0.98–1.52)	1.21 (0.97–1.51)	1.22 (0.98–1.53)	1.22 (0.98–1.53)	1.22 (0.98–1.53)
Female		1.00	1.00	1.00	1.00	1.00	1.00
Age							
6–11		0.79 (0.52–1.19)	0.74 (0.48–1.16)	0.70 (0.44–1.11)	0.69 (0.43–1.10)	0.68 (0.43–1.09)	0.68 (0.43–1.10)
12–23		0.98 (0.69–1.41)	0.93 (0.65–1.34)	0.88 (0.61–1.28)	0.88 (0.61–1.27)	0.88 (0.61–1.28)	0.88 (0.61–1.28)
24–35		0.98 (0.68–1.42)	0.96 (0.66–1.39)	0.88 (0.62–1.29)	0.88 (0.60–1.28)	0.88 (0.61–1.29)	0.88 (0.60–1.28)
36–47		1.24 (0.87–1.78)	1.23 (0.86–1.77)	1.19 (0.83–1.72)	1.20 (0.83–1.73)	1.20 (0.83–1.72)	1.19 (0.83–1.72)
48–59		1.00	1.00	1.00	1.00	1.00	1.00
Multiple births							
Single births		0.88 (0.41–1.90)	0.89 (0.41–1.92)	0.85 (0.40–1.83)	0.89 (0.42–1.90)	0.89 (0.42–1.90)	0.88 (0.41–1.90)
Twins		1.00	1.00	1.00	1.00	1.00	1.00
Preceding birth interval							
< 24		1.20 (0.87–1.65)	1.20 (0.87–1.64)	1.11 (0.73–1.67)	1.10 (0.73–1.66)	1.10 (0.73–1.66)	1.11 (0.74–1.66)
24–47		1.15 (0.85–1.57)	1.15 (0.85–1.57)	1.06 (0.75–1.51)	1.06 (0.74–1.50)	1.05 (0.74–1.50)	1.06 (0.75–1.50)
48+		1.00	1.00	1.00	1.00	1.00	1.00
Recent diarrhea^b^							
No			0.86 (0.63–1.16)	0.84 (0.61–1.14)	0.83 (0.61–1.13)	0.83 (0.61–1.13)	0.83 (0.61–1.13)
Yes			1.00	1.00	1.00	1.00	1.00
Recent fever^c^							
No			1.00 (0.78–1.28)	0.99 (0.77–1.29)	0.99 (0.76–1.29)	0.99 (0.76–1.29)	0.99 (0.76–1.29)
Yes			1.00	1.00	1.00	1.00	1.00
Maternal age							
15–19				0.64 (0.33–1.26)	0.62 (0.32–1.22)	0.62 (0.31–1.22)	0.61 (0.31–1.21)
20–24				1.01 (0.68–1.52)	1.00 (0.67–1.49)	1.00 (0.66–1.49)	0.98 (0.66–1.47)
25–29				0.74 (0.49–1.13)	0.73 (0.48–1.11)	0.73 (0.48–1.12)	0.73 (0.48–1.11)
30–34				0.82 (0.54–1.27)	0.82 (0.53–1.25)	0.82 (0.53–1.27)	0.82 (0.53–1.26)
35–49				1.00	1.00	1.00	1.00
Maternal education levels							
No education				1.16 (0.73–1.84)	1.13 (0.71–1.80)	1.13 (0.71–1.80)	1.10 (0.70–1.74)
Primary				0.98 (0.66–1.45)	0.97 (0.65–1.45)	0.96 (0.65–1.45)	0.95 (0.64–1.41)
Secondary and above				1.00	1.00	1.00	1.00
Maternal body mass index^d^							
Underweight				3.40 (1.93–5.97)*	3.55 (2.03–6.20)*	3.52 (2.01–6.14)*	3.52 (2.02–6.13)*
Optimal weight				1.54 (1.03–2.32)*	1.57 (1.05–2.34)*	1.57 (1.05–2.35)*	1.56 (1.04–2.34)*
Overweight/obesity				1.00	1.00	1.00	1.00
Wealth index							
Poorest				1.74 (1.00–3.03)*	1.76 (1.01–3.07)*	1.77 (1.01–3.14)*	1.76 (0.95–3.24)
Poorer				1.47 (0.84–2.55)	1.46 (0.83–2.56)	1.48 (0.83–2.63)	1.46 (0.79–2.71)
Middle				1.37 (0.76–2.47)	1.35 (0.75–2.45)	1.36 (0.75–2.48)	1.34 (0.70–2.55)
Richer				1.17 (0.64–2.12)	1.17 (0.65–2.14)	1.19 (0.65–2.18)	1.17 (0.62–2.20)
Richest				1.00	1.00	1.00	1.00
No of under-5-year children							
≤ 1				0.57 (0.38–0.91)*	0.58 (0.38–0.90)*	0.59 (0.38–0.90)*	0.59 (0.38–0.90)*
2–3				0.51 (0.34–0.77)*	0.51 (0.34–0.76)*	0.51 (0.34–0.76)*	0.51 (0.34–0.76)*
≥ 4				1.00	1.00	1.00	1.00
Amount of media exposure^e^							
0				3.02 (0.81–11.27)	3.08 (0.82–11.58)	3.14 (0.84–11.81)	3.17 (0.84–11.92)
1				2.73 (0.72–10.30)	2.76 (0.73–10.51)	2.81 (0.74–10.69)	2.89 (0.76–11.00)
2				1.39 (0.33–5.08)	1.43 (0.34–6.07)	1.45 (0.35–6.01)	1.47 (0.35–6.18)
3				1.00	1.00	1.00	1.00
Source of drinking water							
Unimproved					1.31 (0.97–1.77)	1.31 (0.97–1.77)	1.31 (0.97–1.78)
Improved^f^					1.00	1.00	1.00
Sanitation facility							
Unimproved					0.77 (0.56–1.05)	0.77 (0.56–1.05)	0.76 (0.55–1.04)
Improved^g^					1.00	1.00	1.00
Place of delivery							
Home and other						1.12 (0.76–1.66)	1.13 (0.77–1.67)
Hospital/institution^h^						1.00	1.00
Perceived distance to health facility							
Perceived problem						1.78 (0.84–1.39)	1.08 (0.84–1.39)
Not perceived problem						1.00	1.00
Place of residence							
Urban							0.94 (0.51–1.72)
Rural							1.00
Geographical region							
Northern							0.88 (0.60–1.30)
Central							0.87 (0.68–1.12)
Southern							1.00

*P < 0.05; ^a^Proportion of births with a reported birth weight < 2500 g regardless of gestational age; ^b^ passage of three or more loose or liquid stools during a 24-h period; ^c^self-reports by mothers about symptoms that had occurred within 2 weeks prior to the survey; ^d^ weight in kilograms divided by the square of his height in meters (kg/m^2^); ^e^frequency of reading newspaper or magazine, frequency of listening to radio, frequency of watching television.; ^f^improved drinking water (piped water into dwelling, piped water to yard/plot, public tap or standpipe, tubewell or borehole, protected dug well, protected spring and rainwater); ^g^improved sanitation (flush toilet, piped sewer system, septic tank, flush/pour flush to pit latrine, ventilated improved pit latrine, pit latrine with slab, and composting toilet); ^h^government hospital, government health center, government health post/outreach, other public sector, private hospital/clinic, CHAM/MISSION hospital, CHAM/MISSION health Center, BLM, other private medical sector

**Table 4:** Multivariate logistic analysis of the association of low birthweight with childhood wasted while controlling for several covariates

Variables	Model I	Model II	Model III	Model IV	Model V	Model VI	Model VII
	COR 95% (CI)	aOR 95% (CI)	aOR 95% (CI)	aOR 95% (CI)	aOR 95% (CI)	aOR 95% (CI)	aOR 95% (CI)
Birth weight							
Low birthweight^a^	2.42 (1.38–4.25)*	2.63 (1.48–4.67)*	2.63 (1.48–4.69)*	2.54 (1.41–4.56)*	2.53 (1.41–4.57)*	2.56 (1.42–4.63)*	2.53 (1.40–4.57)*
Normal and above	1.00	1.00	1.00	1.00	1.00	1.00	1.00
Sex of the child							
Male		1.52 (0.95–2.45)	1.49 (0.93–2.39)	1.53 (0.94–2.52)	1.56 (0.95–2.56)	1.56 (0.95–2.57)	1.57 (0.95–2.59)
Female		1.00	1.00	1.00	1.00	1.00	1.00
Age							
6–11		1.94 (0.87–4.37)	1.46 (0.65–3.26)	1.18 (0.50–2.83)	1.14 (0.48–2.75)	1.14 (0.47–2.74)	1.07 (0.43–2.62)
12–23		1.93 (0.89–4.18)	1.43 (0.66–3.11)	1.27 (0.56–2.93)	1.27 (0.55–2.90)	1.27 (0.55–2.91)	1.25 (0.55–2.88)
24–35		1.15 (0.54–2.47)	0.96 (0.45–2.07)	0.94 (0.42–2.07)	0.93 (0.42–2.07)	0.92 (0.41–2.06)	0.91 (0.41–2.03)
36–47		1.07 (0.47–2.43)	0.99 (0.43–2.25)	0.99 (0.43–2.32)	0.99 (0.42–2.32)	0.98 (0.42–2.31)	0.95 (0.41–2.23)
48–59		1.00	1.00	1.00	1.00	1.00	1.00
Multiple births							
Single births		9.62 (2.15–43.0)*	9.43 (2.07–43.0)*	12.8 (2.59–63.1)*	13.5 (2.73–66.4)*	13.0 (2.67–63.6)*	13.2 (2.69–64.4)*
Twins		1.00	1.00	1.00	1.00	1.00	1.00
Preceding birth interval							
< 24		1.44 (0.81–2.60)	1.44 (0.81–2.57)	1.04 (0.50–2.17)	1.02 (0.49–2.12)	1.02 (0.49–2.12)	1.08 (0.51–2.28)
24–47		1.07 (0.58–1.94)	1.07 (0.58–1.96)	0.87 (0.41–1.86)	0.85 (0.40–1.82)	0.85 (0.40–1.81)	0.91 (0.42–1.96)
48+		1.00	1.00	1.00	1.00	1.00	1.00
Recent diarrhea^b^							
No			0.58 (0.37–0.93)*	0.59 (0.37–0.96)*	0.59 (0.36–0.95)*	0.59 (0.37–0.95)*	0.59 (0.36–0.97)*
Yes			1.00	1.00	1.00	1.00	1.00
Recent fever^c^							
No			0.51 (0.32–0.82)*	0.47 (0.29–0.78)*	0.48 (0.29–0.78)*	0.48 (0.29–0.79)*	0.48 (0.29–0.80)*
Yes			1.00	1.00	1.00	1.00	1.00
Maternal age							
15–19				1.24 (0.41–3.72)	1.21 (0.40–3.59)	1.24 (0.41–3.71)	1.11 (0.37–3.36)
20–24				0.90 (0.42–1.93)	0.90 (0.42–1.90)	0.91 (0.43–1.94)	0.83 (0.38–1.78)
25–29				0.94 (0.45–1.99)	0.93 (0.44–1.95)	0.94 (0.44–1.99)	0.92 (0.43–1.97)
30–34				0.55 (0.24–1.28)	0.54 (0.23–1.25)	0.55 (0.23–1.28)	0.52 (0.22–1.22)
35–49				1.00	1.00	1.00	1.00
Maternal education levels							
No education				0.70 (0.27–1.78)	0.66 (0.26–1.72)	0.66 (0.26–1.72)	0.65 (0.23–1.82)
Primary				0.80 (0.40–1.59)	0.79 (0.39–1.55)	0.80 (0.39–1.61)	0.85 (0.39–1.83)
Secondary and above				1.00	1.00	1.00	1.00
Maternal body mass index^d^							
Underweight				5.00 (1.64–15.2)*	5.28 (1.68–16.5)*	5.21 (1.67–16.2)*	5.45 (1.71–17.3)*
Optimal weight				2.26 (0.92–5.54)	2.30 (0.93–5.70)	2.25 (0.91–5.61)	2.40 (0.97–5.99)
Overweight/obesity				1.00	1.00	1.00	1.00
Wealth index							
Poorest				0.64 (0.25–1.67)	0.65 (0.25–1.66)	0.63 (0.24–1.68)	0.91 (0.39–2.20)
Poorer				0.45 (0.18–1.80)	0.45 (0.18–1.76)	0.43 (0.17–1.12)	0.63 (0.27–1.47)
Middle				0.62 (0.23–1.66)	0.61 (0.23–1.66)	0.60 (0.22–1.67)	0.86 (0.35–2.08)
Richer				0.65 (0.24–1.75)	0.66 (0.25–1.75)	0.64 (0.23–1.78)	0.85 (0.36–2.04)
Richest				1.00	1.00	1.00	1.00
No of under-5-year children							
≤ 1				0.40 (0.18–0.88)*	0.39 (0.18–0.86)*	0.39 (0.18–0.87)*	0.40 (0.18–0.61)*
2–3				0.44 (0.21–0.96)*	0.44 (0.21–0.94)*	0.44 (0.21–0.94)*	0.44 (0.21–0.94)*
≥ 4				1.00	1.00	1.00	1.00
Amount of media exposure^e^							
0				1.15 (0.16–8.05)	1.17 (0.17–8.25)	1.08 (0.15–7.58)	1.11 (0.15–7.58)
1				0.76 (0.11–5.23)	0.78 (0.11–5.34)	0.71 (0.10–4.91)	0.76 (0.11–5.24)
2				0.55 (0.06–4.94)	0.56 (0.06–5.06)	0.53 (0.06–4.89)	0.51 (0.06–4.54)
3				1.00	1.00	1.00	1.00
Source of drinking water							
Unimproved					1.40 (0.76–2.56)	1.38 (0.76–2.53)	1.44 (0.79–2.65)
Improved^f^					1.00	1.00	1.00
Sanitation facility							
Unimproved					0.72 (0.42–1.25)	0.73 (0.42–1.25)	0.72 (0.42–1.24)
Improved^g^					1.00	1.00	1.00
Place of delivery							
Home and other						1.08 (0.53–2.23)	1.06 (0.51–2.20)
Hospital/institution^h^						1.00	1.00
Distance to health facility							
Perceived problem						0.83 (0.50–1.39)	0.81 (0.48–1.36)
Not perceived problem						1.00	1.00
Place of residence							
Urban							2.21 (1.01–4.84)*
Rural							1.00
Geographical region							
Northern							0.65 (0.30–1.41)
Central							0.70 (0.40–1.22)
Southern							1.00

* < 0.05; ^a^Proportion of births with a reported birth weight < 2500 g regardless of gestational age; ^b^passage of three or more loose or liquid stools during a 24-h period; ^c^self-reports by mothers about symptoms that had occurred within 2 weeks prior to the survey; ^d^weight in kilograms divided by the square of his height in meters (kg/m^2^); ^e^frequency of reading newspaper or magazine, frequency of listening to radio, frequency of watching television.; ^f^improved drinking water (piped water into dwelling, piped water to yard/plot, public tap or standpipe, tubewell or borehole, protected dug well, protected spring and rainwater); ^g^improved sanitation (flush toilet, piped sewer system, septic tank, flush/pour flush to pit latrine, ventilated improved pit latrine, pit latrine with slab, and composting toilet); ^h^government hospital, government health center, government health post/outreach, other public sector, private hospital/clinic, CHAM/MISSION hospital, CHAM/MISSION health Center, BLM, other private medical sector

## Discussion

It is known that childhood undernutrition is a primary cause of mortality and morbidity in millions of preschool-aged children worldwide [1–3, 35]. Whilst many studies have examined the levels and predictors of childhood undernutrition in different settings [2, 36, 37], very limited empirical evidence has been reported on the variation of childhood undernutrition across the birth weight status of preschool children. For example, studies in Malawi found that a series of socio-demographic, comorbidities and nutritional characteristics were significantly associated with childhood undernutrition [2, 38–40]. Unfortunately, none of these studies have examined the independent association of birth weight on nutritional status among children below 5 years of age, despite its detrimental consequences. Hence, the present study was conducted to investigate how and to what extent LBW is associated with undernutrition among preschool-aged children in Malawi while adjusting for other known risk factors.

The current study has revealed a strong positive association between LBW and undernutrition among preschool-aged children in Malawi. For instance, the risk of being stunted (57%), underweight (15%) and, wasted (51%) in the course of the early childhood years were found to be higher in children with LBW than in those with normal birth weights even after controlling for other known risk factors for childhood undernutrition in models. Thus, children who have LBW are at increased risk to remain undernourished in the course of the early years of their childhood even after controlling for the child’s biological, child’s health, maternal/households, household environmental, healthcare utilization and community characteristics. In this study, the pragmatic relationship between birth weight and childhood undernutrition is in line with the results of studies conducted elsewhere [3, 23–25, 41].

For instance, in Malawi increased odds of stunting and underweight were found among children with perceived small sizes at birth than average birth sizes [3]. Similarly, in a study of infant growth patterns and their relations to birth weight in Bangladesh found that birth weight was the most essential predictor of succeeding growth status for the period of infancy [41]. Several epidemiological studies have reported that children with perceived small sizes at birth are often born from households with low socioeconomic status and poor maternal health conditions (i.e. maternal undernutrition) [3, 41].

In Brazil, a study of LBW where only low-income families were included found that the LBW children resided in households with poor environments, fewer resources, and mothers with less education than their average birth weights counterparts [42]. Prior researchers have also suggested that children born with a LBW are at increased risk for neurological aberrations and delayed growth and development in their early years [43]. Additionally, the relationship between LBW and childhood undernutrition may be mediated by increased morbidity (infection) in LBW children [42, 44]. It is well documented that malnutrition is the major cause of the immunodeficiency thus increasing susceptibility to infections whilst infections exacerbate malnutrition by reducing appetite, reducing absorption, inducing catabolism, and increasing excretion due to vomiting and diarrhea [45, 46]. Previously it has been reported that the very-low-birth-weight infants are at high risk for developing an infection during the antenatal, prenatal, and postnatal periods [47]. Hence, all of these conditions can negatively affect child growth patterns.

### Strengths and weakness

The strength of the present study is the use of the nationally representative dataset which means the results can be generalized at Malawian setting. However, the potential limitations are (1) the use of cross-sectional study design, thus cannot permit us to draw causal inferences and (2) the use of secondary analysis did not permit the inclusion of relevant factors to control for poor pregnancy conditions.

### Policy implication

This study is one of its kind to investigate the independent association of birth weight with childhood undernutrition in Malawi. In particular, the risk of growth and development impairment in the early childhood years were found to be higher in children with LBW than in those with normal birth weights even after controlling for other known risk factors for childhood undernutrition in models. The finding of the current study seems to be an alarm call for all policymakers involved in the development of childhood nutrition policies in Malawi. Thus, the nutrition events throughout the life cycle should not be neglected. Pregnant women should be encouraged to follow good health habits such adequate intake of good nutritious diets, preconception weight should be monitored, they should be discouraged from smoking, and they should be treated for anemia, malaria, and any intrauterine infection so as to minimize LBW deliveries.

## Conclusion

The current study has revealed that the prevalence of undernutrition remains unacceptably high in Malawi, despite numerous interventions that are being implemented. The growth and development of the child are significantly predisposed by several factors. In this study, LBW babies were strongly associated with an increased risk of undernutrition. Therefore, in addition to breastfeeding, complementary feeding practices, disease prevention and case management, improved household environments, maternal education, parental behavior that previous studies suggested, interventions that aim at improving the growth and development of preschool-aged children should begin right in utero in order to avoid IUGR which result into LBW.

## Additional files

Additional file 1: **Table S1.** Results of multicollinearity testing for undernutrition in Malawi (DOC 43 kb)

Additional file 2: **Figure S1.** Fit Diagnostics for Childhood Undernutriton in Malawi (a) Stunting, (b) Underweight, (c) Wasted (JPG 1607 kb)

## Data Availability

The datasets generated and/or analyzed during the current study are available in the DHS Program repository, http://dhsprogram.com/data/available-datasets.cfm.

## Abbreviations

AOR: Adjusted Odds Ratio
CDC: Centers for Disease Control
CFR: Code of Federal Regulations
IRB: Institutional Review Board
IUGR: Intrauterine Growth Retardation
LBW: Low Birth Weight
MDHS: Malawi Demographic and Health Survey
NC: New York City
NHSRC: National Health Sciences Research Committee
PROC: Procedure
SAS: Statistical Analysis Software
SD: Standard Deviations
UNICEF: United Nations Children’s Fund
USA: United States of America
WHO: World Health Organization
