# Supplementary material for: Association of low birth weight with undernutrition in preschool-aged children in Malawi
Source: Nutr J. 2019 Sep 2;18:51. doi: 10.1186/s12937-019-0477-8 (PMC6719380; doi:10.1186/s12937-019-0477-8)
Supplement: Supplementary file 1 — Table S1. Results of multicollinearity testing for undernutrition in Malawi (DOC 43 kb) [file 12937_2019_477_MOESM1_ESM.doc]

| Additional file 1 Table S1. Results of multicollinearity testing for undernutrition in Malawi | | | |
| --- | --- | --- | --- |
| Variable | | Tolerance | VIF^a^ |
|  | Low birth weight^b^ | 0.97 | 1.03 |
|  | Sex of the child | 0.99 | 1.01 |
|  | Age | 0.86 | 1.16 |
|  | Multiple births | 0.94 | 1.06 |
|  | Preceding birth interval (months) | 0.66 | 1.51 |
|  | Recent diarrhea^c^ | 0.89 | 1.13 |
|  | Recent fever^d^ | 0.94 | 1.06 |
|  | Maternal age (years) | 0.63 | 1.60 |
|  | Maternal education levels | 0.78 | 1.28 |
|  | Maternal body mass index^e^ | 0.94 | 1.07 |
|  | Wealth index | 0.66 | 1.52 |
|  | No of under-5-year children | 0.92 | 1.09 |
|  | Amount of media exposure^f^ | 0.79 | 1.27 |
|  | Source of drinking water | 0.94 | 1.06 |
|  | Sanitation facility | 0.95 | 1.05 |
|  | Place of delivery | 0.97 | 1.03 |
|  | Distance to health facility | 0.90 | 1.11 |
|  | Place of residence | 0.77 | 1.30 |
|  | Geographical region | 0.95 | 1.05 |
| *^a^Variance Inflation ^b^Proportion of births with a reported birth weight <2500 grams regardless of gestational age; ^c^passage of three or more loose or liquid stools during a 24-h period; ^d^self-reports by mothers about symptoms that had occurred within 2 weeks prior to the survey; e, weight in kilograms divided by the square of his height in meters (kg/m^2^); ^f^frequency of reading newspaper or magazine, frequency of listening to radio, frequency of watching television.; ^g^improved drinking water (piped water into dwelling, piped water to yard/plot, public tap or standpipe, tubewell or borehole, protected dug well, protected* | | | |
