# Supplementary figures and images for: Association of low birth weight with undernutrition in preschool-aged children in Malawi
Source: Nutr J. 2019 Sep 2;18:51. doi: 10.1186/s12937-019-0477-8 (PMC6719380; doi:10.1186/s12937-019-0477-8)

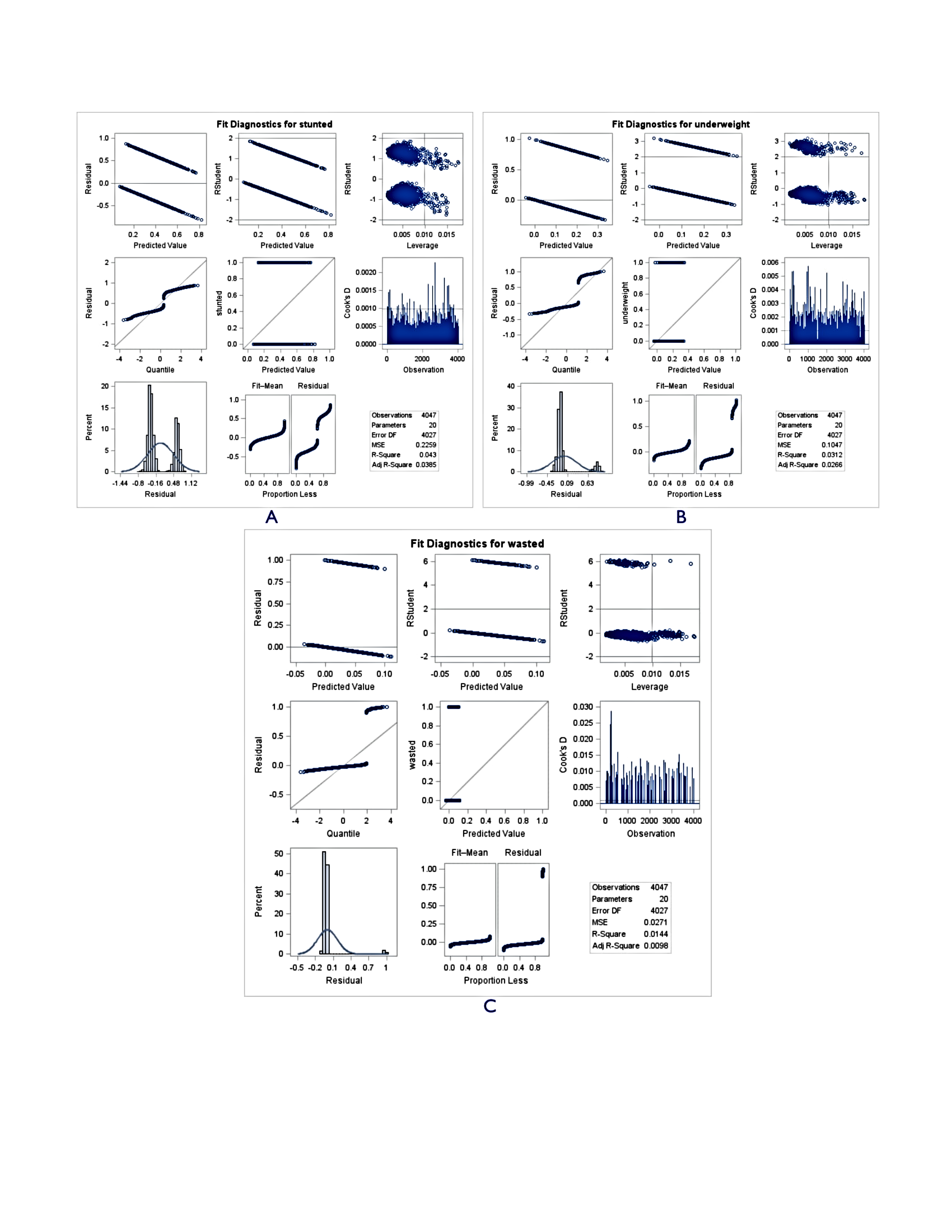

Supplement: Supplementary file 2 — Figure S1. Fit Diagnostics for Childhood Undernutriton in Malawi (a) Stunting, (b) Underweight, (c) Wasted (JPG 1607 kb) [file 12937_2019_477_MOESM2_ESM.jpg]
